# Inebilizumab, a B Cell-Depleting Anti-CD19 Antibody for the Treatment of Autoimmune Neurological Diseases: Insights from Preclinical Studies

**DOI:** 10.3390/jcm5120107

**Published:** 2016-11-24

**Authors:** Ding Chen, Sandra Gallagher, Nancy L. Monson, Ronald Herbst, Yue Wang

**Affiliations:** 1 Department of Neurology and Neurotherapeutics, University of Texas Southwestern Medical Center, Dallas, TX 75390, USA; ding.chen@ustouthwestern.edu (D.C.); nancy.monson@utsouthwestern.edu (N.L.M.); 2 Department of Project Management, MedImmune, Gaithersburg, MD 20878, USA; gallaghers@medimmune.com; 3 Department of Oncology Research, MedImmune, Gaithersburg, MD 20878, USA; herbstro@medimmune.com

**Keywords:** CD19, B-cell depletion, autoimmunity, multiple sclerosis, neuromyelitis optica

## Abstract

Exaggerated or inappropriate responses by B cells are an important feature in many types of autoimmune neurological diseases. The recent success of B-cell depletion in the treatment of multiple sclerosis (MS) has stimulated the development of novel B-cell-targeting therapies with the potential for improved efficacy. CD19 has emerged as a promising target for the depletion of B cells as well as CD19-positive plasmablasts and plasma cells. Inebilizumab (MEDI-551), an anti-CD19 antibody with enhanced antibody-dependent cell-mediated cytotoxicity against B cells, is currently being evaluated in MS and neuromyelitis optica. This review discusses the role of B cells in autoimmune neurological disorders, summarizes the development of inebilizumab, and analyzes the recent results for inebilizumab treatment in an autoimmune encephalitis mouse model. The novel insights obtained from these preclinical studies can potentially guide future investigation of inebilizumab in patients.

## 1. Introduction

Many neurological diseases, such as multiple sclerosis (MS), neuromyelitis optica (NMO), myasthenia gravis (MG), and autoantibody-mediated encephalitis, arise from an abnormal immune response toward nervous tissues. In MS, the dysregulated immune system attacks the myelin sheath that covers the nerve fibers, disrupting communication between the brain and the body [1]. In most cases of NMO, autoantibodies against the water channel protein aquaporin-4 (AQP4) induce astrocyte injury, leading to inflammation and demyelination of the optical nerve and the spinal cord [2]. Patients with MG develop autoantibodies against either the muscle-specific tyrosine kinase or the acetylcholine receptor at the postsynaptic membrane of the neuromuscular junction, causing impaired neuromuscular transmission and weakened muscle strength [3]. Patients with autoantibodies targeting the *N*-methyl-d-aspartate receptor develop a severe form of encephalitis with neuropsychiatric symptoms [4,5]. Although these disorders are distinguished by their manifestations and symptoms, they share underlying defects in the suppression of autoimmune responses mediated by B cells and autoantibodies, resulting in central or peripheral nervous system dysfunction.

Most autoimmune neurological disorders are chronic conditions that potentially cause long-term disability and therefore impose a substantial burden on patients, caretakers, and the health care system. Patients with autoimmune neurological diseases generally respond to therapies that suppress immune system function, but a significant proportion of patients have only incomplete responses. In patients who show significant improvement initially, effects are generally not permanent and disease relapses are frequent. Currently there are no curative treatments for these diseases, and no medications have been approved for the treatment of NMO or autoantibody-mediated encephalitis [1,2,3,4,5].

An understanding of the shared and distinguishing features of autoimmune neurological diseases provides a conceptual framework for the development of novel therapeutic agents. The presence of autoantibodies is a characteristic feature of MS, NMO and MG, and therapies targeting B cells have come to the center stage over the past decade for the treatment of these diseases. Monoclonal antibodies (mAbs) against the human CD20 antigen represent the best-studied B-cell-targeting immunotherapies, and to date, three anti-CD20 antibodies (rituximab, ocrelizumab, and ofatumumab) have been evaluated for the treatment of MS [6]. These anti-CD20 antibodies kill B cells either by inducing B-cell apoptosis or by triggering Fcγ receptor (FcγR)-mediated antibody-dependent cell-mediated cytotoxicity (ADCC) and/or complement-dependent cytotoxicity of targeted B cells. Several large phase 2 and 3 clinical trials testing anti-CD20 therapies in MS, involving thousands of patients altogether, are being conducted [6]. In general, all three of these anti-CD20 antibodies induce substantial depletion of circulating B cells for periods ranging from a few months to a year. In two phase 3 studies in patients with relapsing-remitting MS, ocrelizumab has achieved primary and secondary endpoints: patients treated with ocrelizumab have significant reduction of annualized relapse rates, disability progression, and numbers of inflammatory lesions on magnetic resonance imaging (MRI) [6]. The recent success of anti-CD20 antibodies in the treatment of MS validates the fundamental role of B cells in the pathogenesis of this disease and suggests that B-cell-targeting therapies have the potential to be effective not only in MS but also in other forms of autoimmune neurological diseases. 

## 2. Role of B Cells in Autoimmune Neurological Diseases

Many reviews have provided in-depth discussions of B cells and their role in neurological diseases [2,3,5,7,8,9]. Below we provide a summary of recent progresses.

Autoantibodies are produced primarily by plasma cells, a population of terminally differentiated B cells. Among possible pathogenic B-cell functions, autoantibodies are recognized as the most important, having multiple potential pathogenic properties. For example, in NMO patients, AQP4-immunoglobulin G (IgG) directly binds to AQP4 protein on the surface of astrocytes, thereby inhibiting water flux and contributing to intramyelinic edema in early NMO lesions [10]. In addition, AQP4-IgG, mostly in the form of the IgG1 isotype, has the capacity to activate the complement cascade. Components of complement are detectable in NMO lesions and coincide with IgG deposition, indicating antibody-mediated complement activation [11]. Complement-mediated destruction of astrocytes potentially contributes to the loss of AQP4-expressing cells, a histological feature observed in perivascular lesions in NMO patients [12]. Furthermore, AQP4-IgG stimulates natural killer (NK) and myeloid cells via engagement of IgG receptors, leading to immune-mediated tissue damage. In animal models, infusion of AQP4-IgG produces NMO-like lesions through an NK-cell-mediated mechanism [13]. Importantly, tissue injury mediated by autoantibodies often leads to the production of inflammatory cytokines, some of which, such as interleukin-6 (IL-6), further augment autoantibody secretion [14]. Thus, AQP4-IgG is poised to perpetuate and amplify tissue-damaging inflammatory responses in NMO. 

Autoantibodies also play a deleterious role in the pathogenesis of MG and autoimmune encephalitis. In the majority of MG patients, autoantibodies targeting acetylcholine receptor mediate tissue injury, through multiple possible mechanisms, including: (1) block acetylcholine binding to its receptor; (2) induce acetylcholine receptor endocytosis and degradation; (3) activate complement cascade, resulting in the damage of the postsynaptic membrane [15]. A subset of patients with autoimmune encephalitis develop autoantibodies targeting anti-*N*-methyl-d-aspartate receptor and these autoantibodies can potentially cause neuronal dysfunction and tissue injury [16]. Since autoantibodies can contribute to the diseases through multiple mechanisms, depletion of plasma cells and other autoreactive B cells would curtail the root cause of autoantibody production and might provide better clinical outcome than would blocking effector functions of the autoantibodies themselves.

How B cells contribute to the pathogenesis of MS is still unknown, likely with both antibody-dependent and -independent mechanisms of action contributing. B cells can activate T cells directly because B cells have the capacity to present self-antigens to T cells and multiple co-stimulatory molecules, such as CD80, CD86 and inducible costimulator-ligand are known to be expressed on B cells [17]. There is growing evidence that B cells produce a variety of cytokines that modulate autoimmune responses and inflammation [18]. Li et al. recently identified a population of B cells that produce and secrete the cytokine granulocyte macrophage–colony-stimulating factor (GM-CSF). These B cells, which also produce IL-6 and tumor necrosis factor-α, but not IL-10, were twice as frequent in blood from MS patients than from matched healthy volunteers [19]. Interestingly, GM-CSF^+^ B cells derived from MS patients have pro-inflammatory properties, as evidenced by the finding that these cells could stimulate cytokine responses on macrophages [19]. GM-CSF has been known to be critical for the pathogenesis observed in murine models of MS [20], and it has also been reported that GM-CSF-producing CD4 T cells were significantly increased in MS patients [21]. Therefore, GM-CSF^+^ B and T cells might cooperatively promote the activation of myeloid cells in MS, resulting in chronic inflammation within the central nervous system.

In addition to enhancing immune responses, a subset of B cells termed regulatory B cells (Bregs) can suppress immune responses though inhibitory cytokines such as IL-10, IL-35, and transforming growth factor-β [22,23,24,25]. Recent studies in which the number of Bregs was significantly reduced in MS [26], NMO [27,28], and MG [29,30] have demonstrated the relevance of Bregs in autoimmune neurological diseases. In these reports, a reduction in Bregs generally correlated with more severe disease in patients. In contrast, other investigators have shown normal number and function of Bregs in MS patients [31]. These conflicting data indicate the complex role of Bregs in autoimmune neurological diseases. Because all studies so far have focused on Breg cells in blood, it is critical to investigate Bregs in neurological tissues, particularly from disease lesions, to uncover their role in disease pathology. Studies in which Breg cells are used as a potential cellular therapy for murine autoimmune encephalomyelitis have started, opening a new B-cell-targeting approach for treatment of autoimmune neurologic diseases [32].

B cells also contribute to the formation of ectopic lymphoid follicles in the meninges of MS patients. Ectopic follicles are associated with more aggressive MS and a greater extent of tissue injury in the subjacent cortical regions [33,34]. These atypical lymphoid structures contain B, T, and dendritic cells, all of which form aggregates around a special type of stroma cell called follicular dendritic cells (FDCs) [33,34]. FDCs provide signals that sustain B-cell survival and activation through the B-cell activating factor (BAFF) cytokine and the C-X-C motif chemokine ligand 13 (CXCL13). B cells in the ectopic follicles reciprocally support FDC function and maintenance through the lymphotoxin pathway. Multiple lines of evidence indicate that inside these follicles, B cells undergo activation and differentiate into plasma cells, resulting in the secretion of damaging autoantibodies within the central nervous system (CNS) [35]. Therefore, it is important to understand whether B-cell depletion therapy can directly kill B cells residing in ectopic follicles within the central nervous system.

In summary, there is strong evidence that B cells are a crucial player in autoimmune neurological diseases, contributing to disease onset and exacerbation through multiple pathogenic mechanisms. Effective depletion of B cells, including autoreactive plasma cells and pro-inflammatory B cells, might be the key to the development of curative treatment for B-cell-driven autoimmune neurological diseases. 

## 3. Rationale for Targeting CD19 and the Development of Inebilizumab

Anti-CD20 mAbs efficiently deplete mature naïve and memory B cells [36]. However, B cells in the early developmental stages, as well as the majority of plasma cells, are largely resistant to depletion by anti-CD20 because these B-cell subsets do not express detectable CD20 on the cell surface [36]. In contrast, CD19 has a broader expression during B-cell development than does CD20 [37,38]. In bone marrow, B-cell differentiation starts at the pro-B stage and then progresses through pre-B and immature stages to finally become mature B cells. CD19 expression starts at the pro-B cell stage, whereas CD20 expression is initiated at the immature B-cell stage. In the periphery, both CD19 and CD20 are expressed on transitional, naïve, germinal center, and memory B cells. One major difference is on plasma cells: CD20 is absent on mature plasma cells, whereas CD19 is expressed on the majority of plasma cells in the secondary lymphoid organs (such as spleen and tonsils), on all plasma cells in blood, and on more than 50% of plasma cells in bone marrow [39,40]. In addition, CD19 is exclusively expressed on B cells, whereas in contrast, CD20 is also expressed on a subset of CD4^+^ T cells [41]. Therefore, CD19 has both a broader and a more specific pattern of B-cell expression than does CD20.

Levels of CD19 expression are fairly constant on B cells throughout development, undergoing a two- to threefold increase from immature cells to mature B cells, whereas plasma cells have CD19 levels that are comparable to those on mature B cells [37]. On mature B cells, CD19 is physically associated with CD21, CD81, and interferon-induced transmembrane protein 1 (IFITM1, also called Leu-13), and CD81 is required for CD19 surface expression [37]. CD19 has no known ligand and does not seem to undergo significant internalization or shedding from cell surface. These properties make CD19 an attractive target for antibody-mediated depletion of B cells.

Several anti-CD19 mAbs have been developed as immunotherapies [42]. One of these is inebilizumab (MEDI-551), which was developed at MedImmune and has progressed into clinical trials [43,44]. Inebilizumab was derived from the mouse anti-human mAb HB12b, which had already shown impressive activity in depletion of B cells in transgenic mice carrying the human CD19 gene (hCD19 Tg) [45]. HB12b was extensively engineered to further enhance its drug-like properties as well as its B-cell-depleting activity. First, the coding sequence of the F(ab)2 portion of HB12b underwent a humanization process to decrease potential immunogenicity in patients. Second, the humanized F(ab)2 was affinity optimized to enhance binding to human CD19. Lastly, the Fc portion was switched to human IgG1. The resulting high-affinity hIgG1 anti-CD19 mAb was subsequently produced in an α1,6-fucosyltransferase-deficient chinese hamster ovary cell line (using BioWa Potelligent technology) to generate the fucose-free anti-CD19 antibody inebilizumab [43]. The decision to produce an afucosylated antibody was based on compelling evidence that human IgG1 molecules lacking a core fucose residue in the Fc region bind more tightly to FcγRIIIa, which is an activating IgG receptor expressed on NK cells and on macrophages that mediate the antibody-dependent killing function.

To gain insight into its depletion function in vitro, inebilizumab was compared with its variant, anti-CD19-2, which has the same F(ab)2 sequence as inebilizumab but with intact core fucose in the Fc region [43]. Inebilizumab and anti-CD19-2 bind to CD19 on B cells with the same affinity, but inebilizumab has 10-fold higher binding affinity to FcγRIIIa [43]. Accordingly, inebilizumab has stronger ADCC activity than anti-CD19-2 [43]. Inebilizumab potently depleted CD19-expressing B cells, including primary human B cells, B cell lines derived from multiple tumor types, and neoplastic B cells freshly isolated from patients. Importantly, inebilizumab has demonstrated equal or better activity than rituximab in depletion of human primary B cells in autologous ADCC assays [43]. In addition, inebilizumab showed potent ADCC activity against human in vitro-differentiated and primary plasma cells (Casey K. and Herbst, R. unpublished observation). Thus inebilizumab offers a new approach for the treatment of autoimmune diseases because it can directly deplete plasma cells, which are not directly targeted by anti-CD20 mAbs. Driven by these results, clinical trials of inebilizumab treatment have begun. A phase 1 study of the safety and tolerability of inebilizumab in systemic sclerosis (SSc) has been completed (NCT00946699, www.clinicaltrials.gov), and studies of inebilizumab in MS (NCT01585766) and NMO (NCT02200770) are ongoing. In the meantime, inebilizumab is also being studied for treatment of B-cell-related malignancies (NCT01453205 and NCT01466153).

In the phase 1 clinical trial of inebilizumab in patients with either limited or diffuse cutaneous SSc (NCT00946699), inebilizumab treatment led to a rapid, robust, and durable depletion of B cells in whole blood [44]. This pharmacodynamic effect was accompanied by a rapid and significant reduction of plasma cells in both blood and affected skin, indicating that inebilizumab can deplete plasma cells in circulation and in diseased tissue [44,46]. Inebilizumab-treated patients, but not placebo-treated patients, showed an improvement trend in the Modified Rodnan Skin Score from baseline [44], although this was a small study and the clinical activity of inebilizumab must be confirmed by future studies. In addition, inebilizumab was well tolerated and most treatment-emergent adverse events were mild or moderate in severity. Although levels of pathogenic autoantibodies were not analyzed in this study, levels of total immunoglobulins from the majority of inebilizumab-treated patients were within the normal range during the course of monitoring, except in seven subjects presenting lower-than-normal levels of IgG, immunoglobulin A (IgA), or immunoglobulin M (IgM) [44]. These results suggest that further evaluation of inebilizumab in autoimmune diseases, including the autoimmune neurological disorders MS and NMO, is warranted.

HCD19 Tg mice were chosen as a model system to study in vivo functions of inebilizumab because inebilizumab does not recognize CD19 from rodents or non-human primates [43]. In hCD19 Tg mice, the expression pattern of transgenic hCD19 matches that of endogenous CD19 in humans and mice. Bone marrow B cells, from pro-B cells to mature B cells, as well as all spleen and blood B cells, are positive for hCD19 expression. As in humans, transgenic hCD19 can also be detected on the majority of plasma cells in spleen and on more than 50% of plasma cells in bone marrow [45,47]. Furthermore, the amount of hCD19 on B cells from hCD19 Tg mice is comparable to that on primary human B cells [45]. Since CD19 overexpression may contribute to the development of the autoimmune phenotype, we use heterozygous transgenic mice, which have a B-cell repertoire similar to that of the aged-matched wild-type mice and which show minimum abnormality of immune functions. Therefore, hCD19 Tg mice are a relevant preclinical model for studying inebilizumab.

We have demonstrated that inebilizumab administration in hCD19 Tg mice leads to dose-dependent depletion of B cells in blood and lymphoid tissues: after a 1-week treatment with inebilizumab at doses of ≥0.5 mg/kg, more than 90% of B cells in spleen, bone marrow, and blood were depleted [48]. Consistent with the preceding in vitro studies, treatment with CD19-2 (the fucosylated version of inebilizumab) also resulted in B-cell depletion but with reduced efficacy than inebilizumab [48]. Both intravenous and subcutaneous administration of inebilizumab showed comparable efficacy, and its pharmacokinetic profile was similar to those of other human IgG1 mAbs in mice [48].

To compare the pharmacodynamic effects of inebilizumab and rituximab in mice, human CD19-CD20 double transgenic (hCD19/20 Tg) mice were analyzed [43]. Treatment with either inebilizumab or rituximab revealed three main differences [43]. First, both macrophages and complement contributed to B-cell depletion mediated by rituximab, whereas macrophages but not complement mediated inebilizumab B-cell depletion. Second, inebilizumab depleted significantly more bone marrow B cells than did rituximab. Third, the duration of depletion was longer after inebilizumab treatment than after rituximab treatment, probably because inebilizumab is able to deplete early progenitor B cells in bone marrow in hCD19/20 Tg mice. Combined treatment with inebilizumab and rituximab further prolonged B-cell depletion, indicating dual B-cell-depleting therapies can potentially work synergistically [48].

We further evaluated the efficacy of inebilizumab in *Sle1*-hCD19 Tg mice, a well-characterized autoimmune mouse model in which the human *Sle1* gene (a susceptibility locus for systemic lupus erythematosus) and transgenic human CD19 are expressed [47]. In this model, ongoing germinal center responses in secondary lymphoid organs (such as spleen and lymph nodes) are likely to contribute to autoreactive B cells and plasma cells. Inebilizumab elicited rapid and effective B-cell depletion in spleen: more than 90% of germinal center B cells and plasma cells were depleted within the first 2 weeks after a single treatment. In addition, inebilizumab treatment led to a dramatic reduction in CD4^+^ follicular T helper (Tfh) cells, consistent with the important role of B cells for the maintenance of Tfh cells in germinal center responses [49]. Taken together, these findings suggest that inebilizumab may have desirable effects on autoimmune responses because of its broad impact, not only on germinal center B cells and plasma cells, but also (indirectly) on Tfh cells. Consistent with its depletion activity in spleen plasma cells, inebilizumab treatment resulted in a robust reduction of autoantibodies: at 12 weeks, levels of anti-nuclear antibody (ANA) and anti-histone, anti-Sm, anti-ssDNA and anti-dsDNA IgM and IgG antibodies were not only significantly lower than in control mice but were also reduced by ~50% from pretreatment levels in the same animal. Many inflammatory cytokines, such as IL-6, were also dramatically reduced after inebilizumab treatment [47].

In light of the effective depletion of splenic plasma cells in inebilizumab-treated *Sle*-hCD19 Tg mice, an unexpected finding was that bone marrow plasma cells were not depleted even after prolonged treatment with inebilizumab, despite the fact that around half of these cells express hCD19. In contrast, other bone marrow B-cell subsets in the same mice were depleted by 90%, indicating that the local environment inside bone marrow might affect the susceptibility of CD19^+^ cells to inebilizumab-mediated depletion [47]. Further studies are required to understand the apparent protective mechanism in relation to bone marrow plasma cells in this and other disease models. Finally, levels of total IgM, as well as IgA and IgG subclasses, were not changed after treatment with inebilizumab, demonstrating that inebilizumab is effective in reducing the levels of autoantibodies and other inflammatory mediators but has a much smaller effect on total immunoglobulins in serum. 

## 4. Treatment with Inebilizumab in the EAE Model

Experimental autoimmune encephalomyelitis (EAE) has been widely used as a mouse model for studying immune mediated damage to the central nervous system, however, the relevance of the EAE model to the study of human diseases has been debated [50]. Murine EAE model recapitulates many pathological and clinical characteristics of MS, such as mononuclear cell infiltration into the CNS and substantial inflammation-mediated demyelination that results in tissue destruction and axonal loss [51]. In addition, like active CNS lesions in some MS patients, areas of myelin breakdown in EAE also contain B cells, plasma cells, and antibodies [52]. The EAE mouse model has provided considerable insight into the disease mechanisms of MS and other autoimmune neurological disorders and thus has been widely used to study the efficacy of therapeutic agents [52]. 

The classical EAE model is induced by immunization with myelin oligodendrocyte glycoprotein (MOG) peptide 35-55. This EAE model is B-cell independent, probably because MOG peptides bind to the major histocompatibility complex II molecules directly on dendritic cells without processing, leading to peripheral activation of encephalitogenic T cells. In this model, MOG-specific B cells are not activated and do not contribute to the disease [53]. In an alternative model of EAE, onset of disease is induced by immunization with recombinant MOG (rMOG) consisting of the 120-amino acid portion of extracellular domain of MOG. In this alternative rMOG EAE model, B cells are absolutely required for development of disease through two major mechanisms: (1) a sub-population of antigen-activated B cells differentiate into plasma cells, resulting in the production of autoantibodies against MOG; and (2) B cells become activated and serve as antigen-presenting cells, promoting differentiation of pro-inflammatory MOG-specific Th1 and Th17 cells [54]. Anti-MOG autoantibodies targeting multiple epitopes of MOG are not only necessary for the initiation of CNS inflammation, but also cause demyelination [54,55], suggesting that this model is particular useful for modeling anti-MOG mediated demyelination in human patients [56]. Since B cells and autoantibodies play an essential role in pathogenesis in the rMOG-induced EAE model, we conclude that the rMOG EAE model, in which B cells are required for disease initiation and progression [54,57], is instructive for modeling the therapeutic effects of B-cell-targeting mAbs in vivo.

In addition to the above immunization induced EAE models, MOG-specific T cell receptor (TCR) transgenic mice spontaneous develop inflammation in the central nervous system [58]. One important model was created, in which T cells carried a T cell antigen receptor transgene specific for MOG35-55 peptide and B cells expressed a B cell antigen receptor (BCR) transgene for MOG protein [59,60]. These double TCR and BCR transgenic mice spontaneously developed autoimmune demyelination in their spinal cords and optic nerves, and therefore termed opticospinal EAE (OSE) mice. The OSE model with overlapping pathological features of human NMO and MS diseases can be particularly useful for studying cooperation between B and T cells in driving autoimmune neurological diseases. However, MOG specific B cells from OSE mice have a fixed immunoglobulin heavy chain and thus the breadth of reactivity of these B cells is limited. In contrast, B cell response in both rMOG EAE model and in human NMO is complex and polyclonal, recognizing both linear and conformation-dependent epitopes.

Anti-CD20 antibodies have shown efficacy in the rMOG EAE model. They rapidly deplete peripheral B cells and ameliorate EAE severity by inhibiting adaptive immune responses such as antigen-specific Th1 and Th17 responses [61,62]. These findings are consistent with a recent study demonstrating that B-cell MHC II expression is pivotal for activation of pro-inflammatory Th1 and Th17 and susceptibility to EAE [57]. However, anti-CD20 antibodies have a limited effect on autoantibody levels, probably because plasma cells express diminished levels, or completely lack expression, of CD20 on their surfaces [61,63,64]. In addition, in the EAE model induced with MOG peptide, anti-CD20 antibodies negatively impact regulatory B-cell responses and enhanced pro-inflammatory monocyte reactivity [65,66]. These data imply that more selective and effective targeting of pathogenic B cells may be required to enhance the safety and effectiveness of CD20 mAbs and of B-cell depletion therapy in general.

Inebilizumab was also tested in the rMOG EAE model in a recent study [56] in which a single administration disrupted EAE development in hCD19 transgenic mice. Specifically, inebilizumab inhibited leukocyte infiltration into the spinal cord and depleted both short-lived and long-lived plasma cells, resulting in a significant reduction of total and MOG-specific antibodies in the periphery and the CNS. Interestingly, regulatory B cells were partially resistant to depletion, whereas antigen-specific regulatory T-cell responses were promoted in inebilizumab-treated animals [63]. These results indicate that inebilizumab effectively disrupts EAE by reducing adaptive immune responses that are known to participate in disease pathogenesis while sparing regulatory mechanisms that have been shown to suppress the disease.

The contribution of plasma cells to MS remains unclear, although the pathological role of autoantibodies certainly implies a role for autoantibody-producing plasma cells [67,68,69]. To investigate the role of plasma cells in the rMOG EAE model, we compared the efficacy of inebilizumab, which would presumably target plasma cells, with that of a potent anti-mouse CD20 mAb (MB20-11), which would be expected to spare plasma cells. In this model, inebilizumab surpassed CD20 mAb in ameliorating EAE progression and leukocyte infiltration into the CNS. As expected, inebilizumab more significantly reduced the frequency of myelin-specific antibody-producing cells in CNS and peripheral tissue, as well as total and autoantibody levels in serum. Interestingly, CD20 mAb-treated animals presented various degrees of disease severities, and EAE severity score positively correlated with frequency of residual autoantibody-secreting plasma cells in the bone marrow of individual mice. We further confirmed that the bone marrow and secondary lymphoid tissues of EAE mice contained a CD19^+^ CD20^−^ plasma cell population that was depleted only by inebilizumab and not by the CD20 mAb [64]. These data together demonstrate that autoantibody-secreting plasma cells, especially long-lived CD19^+^ CD20^‒^ plasma cells in bone marrow, contribute to disease severity in EAE and are effectively targeted by inebilizumab. CD19^+^ CD20^‒^ B cells are also present in the CNS of MS patients [64], and therefore these data support the notion that CD19 mAb may be more effective than CD20-targeting agents for the treatment of MS.

Although inebilizumab has shown the potential to treat a wide range of autoimmune diseases, it is necessary to recognize its possible side effects, including those associated with long-term B-cell depletion. It is well established that long-lived plasma cells are the major source of persistent antibodies that are important for fighting infections [70,71]. Thus, in clinical studies of any B-cell-depleting agent, it is important to monitor changes in pathogenic autoantibodies as well as protective antibodies induced by previous infection and vaccination. In addition, B cells also contribute to the establishment and survival of memory CD4^+^ T cells and previous studies have shown that CD20-mediated B-cell depletion curtails CD4^+^ T-cell memory in a murine model of virus infection [72]. In children and adolescents with autoimmune neurological disorders, severe cytomegalovirus infections were observed in small numbers of patients receiving rituximab treatment [73], indicating that B-cell depletion may carry a risk of virus reactivation. Another potential concern with B-cell depletion therapy is its capacity to deplete regulatory B cells. Although regulatory B cells show some level of resistance to CD19 mAb depletion, they are still depleted after treatment with inebilizumab or anti-CD20 mAb in the EAE model. In light of these potential risks, hCD19 Tg mice were comprehensively evaluated after single and multiple intravenous or subcutaneous administrations of inebilizumab, and no major adverse effects have been observed in these studies so far. In addition, in *Sle1*-hCD19 Tg mice, inebilizumab treatment had only a minimum effect on plasma cells in bone marrow and did not reduce total immunoglobulins in circulation [47]. Clearly, more animal studies and continued surveillance in clinical studies are warrantied to address the possible side effects of CD19-directed approaches.

## 5. Conclusions

The results from animal studies have demonstrated the superior depletion activity of inebilizumab (relative to CD20-targeting mAbs) against a broad range of B-cell subsets, including plasma cells that secrete autoantibodies. Studies in the *Sle1*-hCD19 Tg and EAE models have highlighted the ability of inebilizumab to suppress levels of autoantibodies and inflammatory cytokines and to reduce the number of Tfh cells. These findings provide a relevant mechanistic basis for the development of inebilizumab for treatment of autoimmune neurological diseases such as NMO and MS and suggest that inebilizumab has the potential to provide benefit for patients.

Supported by preclinical results, inebilizumab has progressed into clinical trials in systemic sclerosis, MS, and NMO and has already demonstrated encouraging preliminary results in patients with systemic sclerosis [44]. In addition to the deep insights gained from preclinical models, clinical data for inebilizumab will contribute to our understanding of the clinical and pathological nature of autoimmune neurological diseases and support the hypothesis that depletion of B cells and plasma cells could benefit a broad range of autoantibody-mediated autoimmune diseases. 
